# 1468. Antibiotic Prophylaxis Prior to Dental Procedures for Total Hip Arthroplasty and Total Knee Arthroplasty Patients.

**DOI:** 10.1093/ofid/ofad500.1305

**Published:** 2023-11-27

**Authors:** Samantha Simon, Alya Aziz, Gloria Goden, Brian L Hollenbeck

**Affiliations:** New England Baptist Hospital, Boston, Massachusetts; New England Baptist Hospital, Boston, Massachusetts; New England Baptist Hospital, Boston, Massachusetts; New England Baptist Hospital/ Beth Israel Lahey Health, Boston, Massachusetts

## Abstract

**Background:**

Guidelines no longer recommend use of prophylactic antibiotics for dental procedures after total hip and total knee arthroplasty (THA/TKA), yet many surgeons continue to routinely prescribe antibiotics for this purpose. In a setting where one must consider the cost of antibiotics and consequences of antibiotic resistance, there may be reason to rethink the practice of prescribing antibiotics prior to dental procedures.

**Methods:**

We conducted a retrospective cohort study of 10,899 patients who underwent THA/TKA between 1/1/19 and 12/31/20 with one of the 13 surgeons at a single institution. Patients were excluded if they had prior infection in the same joint. The primary outcome was late-presenting PJI, defined as PJI diagnosis > 90 days after surgery. Patients were designated in the antibiotic group or non-antibiotic group based on their surgeon’s prophylaxis protocol. Dental-associated PJIs were considered if the patient had evidence of dental infection, poor dentition and/or a dental procedure within a few weeks prior to onset of PJI symptoms.

**Results:**

There were 2,872 (26.4%) patients in the no antibiotics group and 8,027 (73.6%) patients in the antibiotics group (prescribed 2000mg of Amoxicillin 30 minutes prior to dental procedures) (Table 1). We identified 27 (0.3%) late presenting PJIs and 4 (0.03%) dental-associated PJIs (Table 2). 3 of the 4 dental-associated PJI were in people who had dental infections, rather than routine dental cleanings. In the multivariable analysis, BMI ≥ 30 (OR 2.7, CI 1.1-6.5) and revision surgery (OR 7.7, CI 3.1-19.4) were the only variables that increased late-presenting PJI risk (Table 3). Age, gender, American Society of Anesthesiology Score, occlusive silver dressing, and prescription of antibiotics were not shown to affect risk of late-presenting PJI.

**Table 1.**
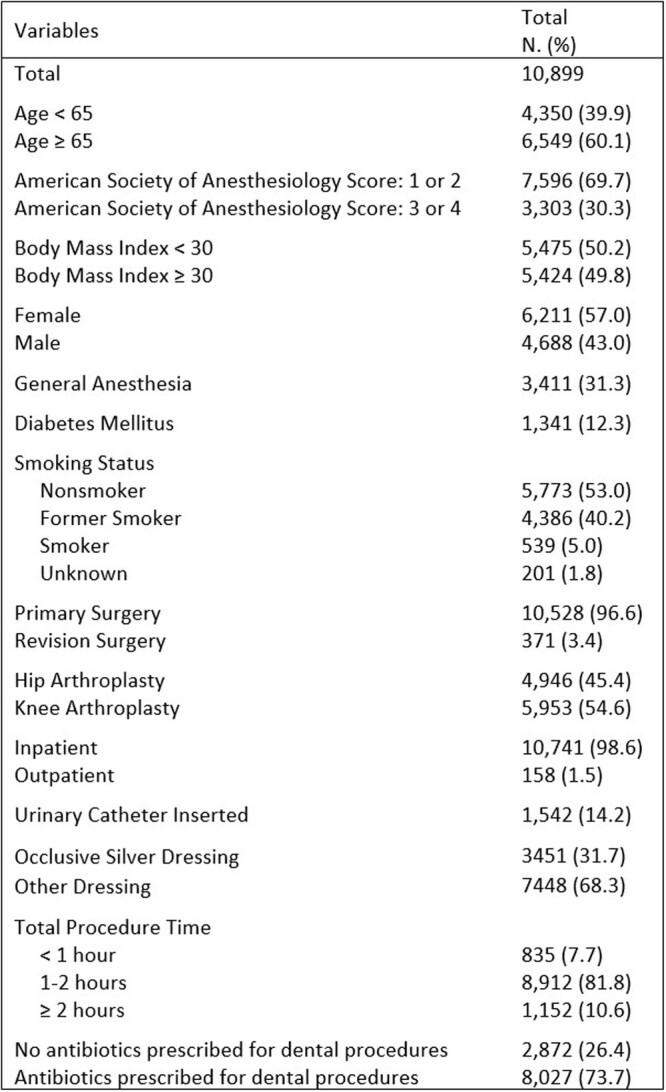
Overview of Patient and Surgical Variables.

**Table 2.**
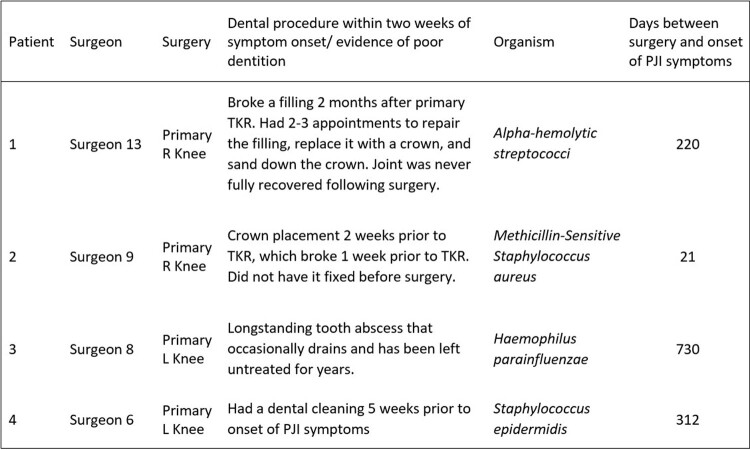
Description of dental-associated prosthetic joint infections.

**Table 3.**
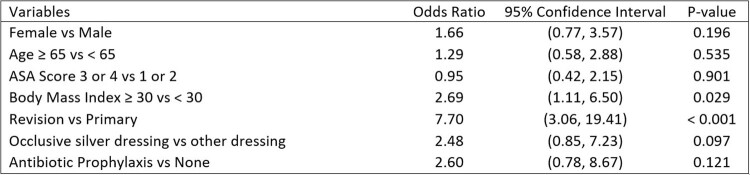
Multivariable Analysis. Adjusted for sex, age, American Society of Anesthesiology Score, body mass index, revision vs primary surgery, and dressing type. Showing odds of developing a late presenting prosthetic joint infection.

**Conclusion:**

In this retrospective cohort study, we found a low rate of late-presenting PJI. Routine prescriptions of antibiotics prior to dental procedures did not alter the risk of late-presenting PJI. In addition, all 4 dental-associated PJIs occurred in patients prescribed antibiotics. These findings demonstrate that antibiotic prophylaxis before dental procedures may not be necessary, but good oral hygiene should be emphasized.

**Disclosures:**

**All Authors**: No reported disclosures

